# Acculturation and the Prevalence of Diabetes in US Latino Adults, National Health and Nutrition Examination Survey 2007–2010

**DOI:** 10.5888/pcd11.140142

**Published:** 2014-10-09

**Authors:** Matthew J. O’Brien, Victor A. Alos, Adam Davey, Angeli Bueno, Robert C. Whitaker

**Affiliations:** Author Affiliations: Victor A. Alos, Temple University and Puentes de Salud Health Center, Philadelphia, Pennsylvania; Adam Davey, Angeli Bueno, Robert C. Whitaker, Temple University, Philadelphia, Pennsylvania.

## Abstract

**Introduction:**

US Latinos are growing at the fastest rate of any racial/ethnic group in the United States and have the highest lifetime risk of diabetes. Acculturation may increase the risk of diabetes among all Latinos, but this hypothesis has not been studied in a nationally representative sample. The objective of this study was to test the hypothesis that acculturation was associated with an increased risk of diabetes in such a sample.

**Methods:**

We conducted a cross-sectional analysis including 3,165 Latino participants in the 2007–2010 National Health and Nutrition Examination Survey. Participants with doctor-diagnosed diabetes and participants without diagnosed diabetes who had glycated hemoglobin (HbA_1C_) values of 6.5% or higher were classified as having diabetes. An acculturation score, ranging from 0 (lowest) to 3 (highest), was calculated by giving 1 point for each of 3 characteristics: being born in the United States, speaking predominantly English, and living in the United States for 20 years or more. Logistic regression was used to determine the association between acculturation and diabetes.

**Results:**

The prevalence of diabetes among Latinos in our sample was 12.4%. After adjusting for sociodemographic factors, the likelihood of diabetes (95% confidence interval [CI]) increased with level of acculturation— 1.71 (95% CI, 1.31–2.23), 1.63 (95% CI, 1.11–2.39), and 2.05 (95% CI, 1.27–3.29) for scores of 1, 2, and 3, respectively. This association persisted after further adjustment for body mass index (BMI), total dietary calories, and physical inactivity.

**Conclusion:**

Acculturation was associated with a higher risk of diabetes among US Latinos, and this risk was only partly explained by BMI and weight-related behaviors. Future research should examine the bio-behavioral mechanisms that underlie the relationship between acculturation and diabetes in Latinos.

## Introduction

Latinos in the United States have a higher lifetime risk of diabetes than those in other racial or ethnic groups (1). The age-standardized prevalence of diabetes among Mexican-American adults — the largest subgroup of US Latinos — is 20.1%, compared with 11.0% for non–Hispanic whites and 18.7% for non–Hispanic blacks (2). The prevalence of diabetes in the United States is expected to increase approximately 200% during the next 40 years, and this growing epidemic will continue to disproportionately affect Latinos (3). The magnitude of the diabetes burden among US Latinos, combined with this population’s rapid growth (4), underscores the relevance of identifying factors related to their diabetes risk.

Acculturation is the process whereby individuals from one culture adopt the behaviors, attitudes, and values of the prevailing culture (5). Acculturation has been measured by using validated instruments that explore this construct in-depth or widely available survey questions that serve as proxy measures for this complex social process (6). Studies have consistently demonstrated that acculturation is associated with worsening diet quality (7) and increased weight (8). Such evidence may suggest that acculturation is also positively associated with diabetes risk among Latinos, considering the role of obesity in the pathogenesis of type 2 diabetes. However, studies examining the relationship between acculturation and diabetes in Latinos have reported conflicting results, some showing that acculturation increases diabetes risk (9–12), and others showing no association (13–15). The lack of consistency in these findings may be due to differences in the study populations, or measurement of the exposure or outcome. The nature of the relationship between acculturation and diabetes in Latinos may have implications for the prevention, clinical care, and self-management of diabetes in this high-risk population.

By using data from the 2007–2010 National Health and Nutrition Examination Survey (NHANES), we examined the association between acculturation and diabetes. We used an acculturation score based on country of birth, predominant spoken language, and years of US residence. We also tested whether this association was mediated by body mass index, diet, and physical activity — factors that are influenced by acculturation and known to affect diabetes risk. Our study is the first to include a nationally representative sample of all US Latinos, whereas previous nationally representative studies have included only Mexican-Americans. Studying this novel population may provide further insight into the relationship between acculturation and diabetes in Latinos.

## Methods

Our analysis of the NHANES data was deemed exempt from review by the Temple University Institutional Review Board. NHANES, which is conducted by the National Center for Health Statistics, uses a complex multistage probability sample designed to be nationally representative of the US civilian, noninstitutionalized population. Beginning in 2007, the NHANES sampling strategy was modified to oversample all Latinos, rather than only Mexican Americans, as in previous cycles (16). This modified sampling strategy allows for reliable national estimates of health indicators for all Latinos from 2007 to 2010, whereas previous cycles could yield reliable estimates only for Mexican Americans (16).

NHANES participants complete a home-based interview and a subsequent physical and laboratory examination in a mobile exam center. Procedures for blood collection and processing are described elsewhere (17). Of the 12,153 participants aged 20 years or older, 3,471 Latinos completed the household interview, 96.1% of whom underwent a physical and laboratory examination. The following numbers of Latino participants had glycemic testing as part of the laboratory examination: glycated hemoglobin (HbA_1c_) (n = 3,185; 92%), fasting plasma glucose (FPG) (n = 1,621; 47%), and oral glucose tolerance test (OGTT) (n = 1,172; 34%).

We included adult Latinos who reported having received a doctor’s diagnosis of diabetes (n = 444), and those without doctor-diagnosed diabetes who had available data for HbA_1c_ (n = 2,721). We excluded participants in whom diabetes prevalence could not be determined — those without previously diagnosed diabetes who were missing HbA_1c_ data (n = 253). This accounted for 7.3% of the total Latino sample during this time period (unweighted). Pregnant women were also excluded (n = 53), leaving a final analytic sample of 3,165 Latino adults.

### Measures

**Diabetes prevalence.** Our binary dependent variable for diabetes included both diagnosed and undiagnosed diabetes. Participants were classified as having previously diagnosed diabetes if they answered affirmatively to the question of whether a doctor or health professional had ever told them that they have diabetes, other than during pregnancy. For those who answered the same question negatively, the presence of undiagnosed diabetes was determined on the basis of their HbA_1c_ value. We chose to use HbA_1c_ as the diagnostic criterion for undiagnosed diabetes because many more participants underwent HbA_1c_ testing than FPG or OGTT. A diagnosis of diabetes was therefore defined by either of the following scenarios: 1) having received a doctor’s diagnosis of diabetes with any HbA_1c_ value or missing data for HbA_1c_ (ie, diagnosed diabetes, n = 444); or 2) never having received a diagnosis of diabetes but having an HbA_1c_ value at or greater than 6.5% (ie, undiagnosed diabetes, n = 134). We considered all other participants to not have diabetes (n = 2,587).

**Acculturation score.** Following the example of a recent study, an acculturation score was developed from 3 commonly used proxies for acculturation — nativity, language spoken at home, and duration of US residence (9). Those born outside the 50 states or District of Columbia were considered foreign-born. Those who spoke “only Spanish” or “more Spanish than English” at home were considered Spanish speakers. Those who spoke “only English,” “more English than Spanish,” or “both equally” were considered English speakers. We constructed a binary variable for duration of US residence based on the cut point of 20 years, found by others to be a time period at which the risk of diabetes increases among immigrants (10). In constructing an acculturation score, each of these variables was treated as follows: US-born (1 point) versus foreign-born (0 points); English-speaking (1 point) versus Spanish-speaking (0 points); and living in the United States for 20 years or more (1 point) versus less than 20 years (0 points). These individual scores were summed to compute the acculturation score, ranging from 0 (lowest) to 3 (highest). We used a summary score, rather than using the individual acculturation variables, because these factors are clustered in unique combinations that may confer an additive risk of diabetes not captured by studying each one separately (9).

**Covariates.** We examined 8 participant characteristics that were considered potential confounders of the relationship between acculturation and diabetes: Hispanic origin (Mexican vs other), age, sex, educational attainment, household income, marital status, insurance status, and having a usual source of medical care. Household income was assessed by the income-to-poverty ratio, calculated by dividing participants’ annual household income by the federal poverty level, and categorized into equal quartiles for those with available data (≤0.84, 0.85–1.45, 1.46–2.59, ≥2.60). A fifth income category was created for participants missing income data (N = 446) and was used in dummy variable regression to estimate subclass means for these individuals. Educational attainment was grouped into the following categories based on common educational milestones and their distribution in the study population: less than 9th grade, 9th to 11th grade, high school, and more than high school. Participants self-reported whether they had insurance coverage and a usual source of medical care. Three other covariates — body mass index (BMI), total dietary calories, and physical inactivity — were considered potential mediators of the relationship between acculturation and diabetes. Participants’ BMI was calculated on the basis of their measured weight and height (kg/m^2^). Total dietary calories (in kcal) were estimated from 24-hour dietary recall interviews and reported as a continuous measure of total energy intake. Those who reported doing no leisure-time physical activity were classified as being physically inactive.

### Data analysis

All analyses used sample weights and were conducted in 2013 using Stata SE (StataCorp), version 12.1. For all statistical testing, we used Stata survey commands, which adjust variance estimates to account for the complex sample design of NHANES. We first used descriptive statistics to characterize the study cohort with respect to the outcome (diabetes), the primary predictor variable (acculturation score), and the covariates. We used χ^2^ tests to examine the bivariate association between 11 covariates and both the prevalence of diabetes and level of acculturation (0–3). By using logistic regression, we calculated the odds of participants’ having diabetes at each level of the acculturation score (1–3) compared with the least acculturated group (0). After calculating the unadjusted odds, these odds were adjusted in successive models, adding groups of potentially confounding variables: model 1 (demographics; age + sex + Hispanic origin); model 2 (structural factors; model 1 + insurance status + usual source of care); and model 3 (socioeconomic status; model 2 + educational attainment, income-to-poverty ratio, and marital status). These socioeconomic variables were conceptually related, and these groupings were made a priori. We added these covariates into successive models to demonstrate their effect on the association between acculturation and diabetes. All covariates were included in multivariable models (Table 1).

**Table 1:** The Prevalence of Diabetes Among US Latino Adults by Sociodemographic Characteristics and Body Mass Index, National Health and Nutrition Examination Survey 2007–2010 (N = 3,165)

Characteristics	N (%)^a^	Diabetes^b^, % (95% CI)	No Diabetes^c^, % (95% CI)	*P* Value^d^
**Hispanic origin**				
Mexican	1,981 (63.0)	12.8 (10.6–15.0)	87.2 (85.0–89.4)	.35
Other	1,184 (37.0)	11.8 (10.0–13.5)	88.2 (86.5–90.0)	
**Age, y**				
20–29	577 (25.7)	1.8 (0.8–2.9)	98.2 (97.1–99.2)	<.001
30–39	564 (25.7)	4.5 (2.8–6.1)	95.5 (93.9–97.2)	
40–49	597 (21.9)	10.5 (6.7–14.3)	89.5 (85.7–93.3)	
50–59	531 (13.1)	27.6 (22.2–33.1)	72.4 (66.9–77.8)	
>60	896 (13.6)	36.1 (31.4–40.7)	63.9 (59.3–68.6)	
**Sex**				
Male	1,498 (52.3)	12.2 (10.6–13.7)	87.8 (86.3–89.4)	.58
Female	1,667 (47.7)	12.7 (10.3–15.1)	87.3 (84.9–89.7)	
**Education**				
<9th grade	982 (26.3)	18.6 (13.9–23.2)	81.4 (76.8–86.1)	<.001
9th–11th grade	614 (20.6)	11.8 (8.6–15.0)	88.2 (85.0–91.4)	
High school	592 (20.5)	9.1 (6.4–11.9)	90.9 (88.1–93.6)	
>High school	969 (32.6)	9.9 (8.0–11.8)	90.1 (88.2–92.0)	
**Income-to-poverty^e^**				
≤0.84	648 (21.7)	10.8 (7.3–14.3)	89.2 (85.7–92.7)	.26
0.85–1.45	679 (21.6)	14.7 (10.8–18.4)	85.3 (81.6–89.2)	
1.46–2.59	691 (22.3)	11.7 (9.5–13.9)	88.3 (86.1–90.5)	
≥2.60	707 (21.9)	11.8 (8.8–14.7)	88.2 (85.3–91.2)	
Missing	440 (12.5)	13.9 (11.4–16.3)	86.1 (83.7–88.6)	
**Marital status**				
Married/partner	1,997 (63.8)	12.3 (10.5–14.0)	87.7 (86.0–89.5)	<.001
Widowed/divorced^f^	683 (16.9)	19.5 (15.4–23.6)	80.5 (76.4–84.6)	
Never married	485 (19.3)	6.8 (4.8–8.8)	93.2 (91.2–95.2)	
**Health insurance**				
Yes	1,854 (53.2)	15.2 (13.5–16.9)	84.8 (83.1–86.5)	<.001
No	1,311 (46.8)	9.3 (7.0–11.5)	90.7 (88.5–93.0)	
**Usual source of care**				
Yes	2,323 (68.1)	15.1 (13.3–16.9)	84.9 (83.1–86.7)	<.001
No	842 (31.9)	6.8 (4.8–8.7)	93.2 (91.3–95.2)	
**Body mass index^g^**				
˂25.0 kg/m^2^	665 (22.9)	4.5 (3.0–6.0)	95.5 (94.0–97.0)	<.001
25.0–29.9 kg/m^2^	1,221 (39.0)	9.6 (7.9–11.2)	90.4 (88.8–92.1)	
≥30.0 kg/m^2^	1,234 (38.1)	19.7 (16.8–22.6)	80.3 (77.4–83.2)	
**Total dietary calories, kcal (mean)^h^**	NA	1,903 (1,258)	2,190 (991)	<.001
**Physically inactive^i^**				
Yes	1,990 (59.3)	15.4 (13.1–17.6)	84.6 (82.4–86.9)	<.001
No	1,175 (40.7)	8.2 (6.7–9.6)	91.8 (90.4–93.3)	

Abbreviations: CI, confidence interval; NHANES, National Health and Nutrition Examination Survey.

a Unweighted sample size and weighted percentage of subjects in each strata of subject characteristic. Where the total does not add to 3,165, subjects were missing data on that characteristic. Weighted percentages are expressed as column percentages, which add to 100% across all strata of the participant characteristics.

b The weighted percentage (95% confidence interval) of participants with diabetes (the prevalence of both diagnosed and undiagnosed diabetes) within each stratum of participant characteristic (ie, row percentage).

c The weighted percentage (95% confidence interval) of participants without diabetes within each stratum of participant characteristic (ie, row percentage).

d *P *values are for χ^2^ tests comparing the prevalence of diabetes across levels of participant characteristics.

e Expressed as the ratio of participants’ household income to the income at the federal poverty level. This variable was divided into equal quartiles for those with available data and included a separate stratum for those with missing data.

f This category includes individuals who reported being separated.

g Body mass index is determined by the following formula [(weight in kg)/(height in m)^2^] using participants’ measured weight and height.

h Expressed as mean (standard deviation) of total dietary calories on the basis of recall data from 3,080 participants. *t* tests were used to test the difference in total dietary calories between those with and without diabetes.

i Those who reported doing no leisure-time physical activity were classified as being physically inactive.

BMI, total dietary calories, and physical inactivity were included in model 4 to examine whether these factors mediated the primary relationship under study. In addition, generalized structural equation modeling (18) was used to test for mediation of the binary response variable by BMI. Because they are tests of nonlinear hypotheses, coefficients for the indirect, direct, and total effects were obtained with standard error estimates derived by using 10,000 bootstrapped resamples following the method of Preacher and Hayes (19).

## Results

Overall, the prevalence of diabetes in our cohort was 12.4%. The prevalence at each acculturation level was as follows: lowest (35.2%), low (21.6%), high (12.4%), and highest (30.9%). Nearly two-thirds of study participants were of Mexican origin, more than half were aged less than 40 years, and nearly one-third achieved more than a high school education (Table 1). There were significant associations between diabetes and participant age, educational attainment, marital status, health insurance, usual source of care, BMI, total dietary calories, and physical inactivity (Table 1). The same participant characteristics, in addition to the income-to-poverty ratio, were significantly associated with acculturation (Table 2). The prevalence of obesity in our sample was 38.1%. BMI had a strong positive association with diabetes prevalence (Table 1). Although the association between BMI and acculturation was significant, the relationship was not linear (Table 2).

**Table 2:** Levels of Acculturation Among US Latino Adults by Sociodemographic Characteristics and Body Mass Index, NHANES 2007–2010

Characteristic	Acculturation Level (score)^a^				*P* Value
	Lowest (0) % (95% CI)^b^	Low (1) % (95% CI)^b^	High (2) % (95% CI)^b^	Highest (3) % (95% CI)^b^	
**Hispanic origin**					
Mexican	35.1 (28.6–41.6)	20.1 (17.4–22.9)	11.5 (8.7–14.0)	33.3 (29.3–37.4)	.21
Other	35.2 (26.5–44.0)	24.1 (20.4–27.7)	14.1 (10.9–17.3)	26.6 (19.7–33.5)	
**Age, y**					
20–29	40.4 (31.7–49.1)	9.2 (6.6–11.8)	10.0 (6.9–13.0)	40.4 (33.5–47.4)	<.001
30–39	46.1 (40.6–51.6)	13.2 (9.0–17.4)	9.6 (7.3–11.9)	31.1 (25.5–36.7)	
40–49	35.2 (28.7–41.7)	27.4 (23.4–31.4)	14.7 (12.0–17.5)	22.7 (17.1–28.3)	
50–59	23.3 (18.7–27.9)	34.7 (29.6–39.9)	13.9 (10.4–17.4)	28.1 (22.7–33.4)	
>60	15.8 (11.7–19.9)	39.0 (32.2–45.9)	17.0 (13.4–20.6)	28.2 (19.6–36.7)	
**Sex**					
Male	36.3 (29.8–42.7)	22.0 (19.4–24.6)	12.4 (10.3–14.5)	29.4 (24.6–34.2)	.26
Female	34.0 (28.7–39.2)	21.1 (18.6–23.7)	12.4 (9.8–14.9)	32.5 (27.9–37.2)	
**Education**					
<9th grade	53.3 (44.2–62.5)	34.2 (27.8–40.4)	5.7 (3.6–7.8)	6.8 (4.3–9.4)	<.001
9th–11th grade	40.0 (33.4–46.7)	19.3 (16.8–21.9)	13.5 (9.3–17.4)	27.2 (21.4–33.1)	
High school	30.3 (22.2–38.3)	17.8 (14.2–21.4)	13.1 (8.8–17.4)	38.8 (31.9–45.8)	
>High school	20.6 (15.7–25.5)	15.4 (11.5–19.3)	16.5 (14.5–18.5)	47.5 (40.1–54.8)	
**Income-to-poverty^c^**					
≤0.84	45.1 (35.2–55.1)	20.6 (16.3–24.9)	9.5 (5.2–13.6)	24.8 (18.9–30.7)	<.001
0.85–1.44	42.9 (33.7–52.1)	25.6 (21.5–29.7)	12.0 (8.3–15.8)	19.5 (13.5–25.5)	
1.45–2.60	32.9 (26.2–39.7)	21.9 (18.2–25.6)	12.6 (9.6–15.5)	32.6 (25.8–39.4)	
≥2.60	11.6 (7.5–15.7)	19.2 (14.6–23.9)	16.5 (13.5–19.4)	52.7 (45.5–60.0)	
Missing	49.8 (39.6–60.0)	20.0 (15.8–24.1)	10.7 (6.4–14.9)	19.5 (11.8–27.3)	
**Marital status**					
Married/partner	36.9 (31.1–42.6)	22.8 (20.5–25.2)	12.1 (10.2–13.9)	28.2 (23.9–32.5)	<.001
Widowed/divorced^d^	27.1 (20.7–33.6)	25.1 (20.1–30.1)	15.0 (12.1–17.8)	32.8 (25.1–40.5)	
Never married	36.6 (28.5–44.7)	14.4 (11.2–17.6)	11.1 (7.2–15.0)	37.9 (31.3–44.5)	
**Health insurance**					
Yes	20.2 (15.6–24.7)	24.2 (21.2–27.3)	16.4 (14.2–18.6)	39.2 (33.9–44.4)	<.001
No	52.2 (43.9–60.5)	18.5 (15.4–21.7)	7.9 (5.6–10.0)	21.4 (16.2–26.7)	
**Usual source of care**					
Yes	27.7 (23.1–32.3)	23.2 (20.5–25.8)	13.9 (11.8–16.0)	35.2 (30.6–39.9)	<.001
No	51.0 (42.0–60.1)	18.3 (15.2–21.3)	9.1 (6.4–11.9)	21.6 (15.7–27.4)	
**Body mass index^e^**					
˂25.0 kg/m^2^	39.4 (33.2–45.6)	16.6 (13.6–19.5)	12.4 (9.9–15.0)	31.6 (25.2–38.0)	<.001
25.0–29.9 kg/m^2^	40.2 (32.5–47.9)	22.6 (19.4–25.7)	11.5 (8.3–14.6)	25.7 (21.1–30.3)	
≥30.0 kg/m^2^	27.4 (22.9–31.9)	23.6 (21.0–26.1)	13.2 (10.9–15.5)	35.8 (30.9–40.9)	
**Total dietary calories (kcal)^f^**	2,134 (898)	2,015 (1,075)	2,131 (1,013)	2,282 (1,131)	<.001
**Physically inactive^g^**					
Yes	37.8 (31.7–43.9)	25.5 (23.1–27.9)	11.3 (9.1–13.4)	25.4 (20.7–30.2)	<.001
No	31.3 (25.4–37.2)	15.9 (12.9–18.9)	14.0 (11.6–16.4)	38.8 (33.3–44.2)	

Abbreviations: CI, confidence interval; NHANES, National Health and Nutrition Examination Survey.

a Acculturation score represents the sum of the following: US-born (1 point) vs foreign-born (0 points); living in the United States for ≥20 years (1 point) vs <20 years (0 points); speaking English at home (1 point) vs Spanish (0 points). Participants who reported speaking “only Spanish” or “more Spanish than English” at home were classified as Spanish-speaking. All others were classified as English-speaking.

b Weighted row percentages (95% confidence interval) at each acculturation score within strata of participant characteristics.

c Expressed as the ratio of participants’ household income to the income at the federal poverty level. This variable was divided into equal quartiles for those with available data and included a separate stratum for those with missing data.

d This category includes individuals who reported being separated.

e Body mass index is determined by the following formula [(weight in kg)/(height in m)^2^] using participants’ measured weight and height.

f Expressed as mean (standard deviation [SD]) of total dietary calories at each acculturation score. *P* value was <.006 using analysis of variance to examine the association of acculturation score with total dietary calories.

g Individuals who reported doing no leisure-time physical activity were classified as being physically inactive.

The unadjusted prevalence of diabetes by acculturation level was as follows: lowest (6.7%), low (20.5%), high (14.5%), and highest (12.5%). After adjusting for Hispanic origin, age, sex, insurance status, usual source of care, educational attainment, household income, and marital status, the likelihood of diabetes increased with the level of acculturation. Compared with those with an acculturation score of 0, the odds of having diabetes were 1.71 (95% confidence interval [CI], 1.31–2.23), 1.63 (95% CI, 1.11–2.39), and 2.05 (95% CI, 1.27–3.29) for those with acculturation scores of 1, 2, and 3, respectively. Adding BMI, total dietary calories, and physical inactivity to the fully adjusted model reduced the magnitude of the association between acculturation and diabetes only marginally, with this association remaining significant (Table 3, Model 4). Bootstrapped mediation testing confirmed that BMI is a partial mediator of this primary relationship, with the following coefficients (95% CIs) for the indirect, direct, and total effects: 0.049 (0.032–0.065), 0.132 (0.041–0.223), and 0.180 (0.084–0.277), respectively.

**Table 3:** Odds of Diabetes Among US Latino Adults Level of Acculturation, National Health and Nutrition Examination Survey 2007–2010^a^

Acculturation Score	Model 1^b^ OR (95% CI)	Model 2^c^ OR (95% CI)	Model 3^d^ OR (95% CI)	Model 4^e^ OR (95% CI)
0	1 [Reference]	1 [Reference]	1 [Reference]	1 [Reference]
1	1.67 (1.31–2.12)	1.69 (1.28–2.22)	1.71 (1.31–2.23)	1.58 (1.19–2.11)
2	1.40 (1.02–1.93)	1.44 (1.02–2.02)	1.63 (1.11–2.39)	1.61 (1.08–2.38)
3	1.61 (1.12–2.31)	1.63 (1.03–2.59)	2.05 (1.27–3.29)	2.03 (1.27–3.23)

Abbreviations: CI, confidence interval; OR, odds ratio; NHANES, National Health and Nutrition Examination Survey.

a The odds of diabetes are presented for each successive acculturation score compared with the least acculturated group (score of 0).

b Adjusted for age + sex + Hispanic origin.

c Adjusted for Model 1 variables + insurance status + usual source of care.

d Adjusted for Model 2 variables + income–to–poverty ratio + education + marital status.

e Adjusted for Model 3 variables + body mass index + total dietary calories + physical inactivity to assess for potential mediation.

We conducted additional regression analyses for which results are not shown. When each proxy measure of acculturation was examined in a regression model without the other 2, each was associated with a significantly increased risk of diabetes after controlling for covariates.

## Discussion

In a nationally representative sample of US Latinos, the risk of diabetes was higher in those who were more acculturated, even after adjusting for demographic factors and socioeconomic status. The association between acculturation and increased diabetes risk remained significant after adjusting for BMI and weight-related behaviors in multivariable models. Mediation testing revealed that BMI only partially mediated this association between acculturation and diabetes.

This is the first study to examine the relationship between acculturation and diabetes in a nationally representative sample of all US Latinos. Previous NHANES studies on this topic did not involve a nationally representative sample of all Latinos because such a sample was assembled for the first time during the 2007–2010 waves of data collection. Combining all Latinos into 1 group presents both strengths and weaknesses when compared with the existing literature on acculturation and diabetes. Previous studies have documented differences in diabetes rates by Latino subgroup (20), which may provide an argument against aggregating all Latinos in one group. On the other hand, many hospital and clinic systems, government organizations, and other health care stakeholders report health data by Hispanic ethnicity, rather than by individual Latino subgroup. Therefore, many consumers of health data may find a sample of all Latinos more useful in representing their diverse Latino populations in many US communities (21).

Another strength of our study is that we examined undiagnosed diabetes, rather than only including those with doctor-diagnosed diabetes. An HbA_1c_ value of 6.5% or higher was used to classify those with undiagnosed diabetes — an approach that has been formally recommended for diagnosing diabetes by both national and international authorities and is commonly used for that purpose in clinical practice (22). Because those with undiagnosed diabetes constituted almost one-quarter of our sample with diabetes, previous studies that did not examine undiagnosed diabetes or used a different HbA_1c_ cut-off may have been biased by misclassification (11,12).

The cross-sectional nature of this analysis precludes drawing causal inferences about the relationship between acculturation and diabetes. NHANES does not collect data on whether individuals have type 1 or type 2 diabetes. However, on the basis of other studies, more than 90% of cases in NHANES are likely to be type 2 diabetes (23). Using HbA_1c_ as the diagnostic criterion for undiagnosed diabetes limits direct comparison of these findings to other studies that used different diagnostic tests. Although Latinos have higher HbA_1c_ levels than non–Hispanic whites (24), this difference would not be likely to affect the associations we found in our study between acculturation and diabetes among Latinos. Our acculturation score used the 3 most widely used proxy measures for acculturation (5), but the score does not assess all aspects of acculturation, some of which may be related to the risk of diabetes. For example, NHANES does not assess cognitive, emotional, or structural factors that are part of the acculturation process, and some detailed questions in NHANES on language use were not included in the 2007–2010 surveys. More complex measures including other dimensions of the acculturation experience have been developed, but are not available in NHANES or other national data sets.

Our findings are consistent with those of 4 other studies showing that the risk of diabetes increases with acculturation among Latinos (9–12). Only one of these other studies was nationally representative (11), and another found this risk only among Latinos who were not of Mexican ancestry (9). Other studies have found no significant association between acculturation and diabetes among Latinos (13–15). Our findings may have differed from those of other studies using NHANES data that only included Mexican Americans (14,15). In addition, conflicting reports about acculturation and diabetes in US Latinos may reflect the changing nature of this relationship over time as diabetes has become more common in Latin America and is present in more Latinos before they leave their home countries (25).

A novel finding in this study is that BMI and weight-related diet and physical activity behaviors explain only a small proportion of the relationship between acculturation and diabetes risk in Latinos. Like other covariates in our analysis, the association between BMI and acculturation was significant but nonlinear, which may partly explain its small effect on our multivariate mediation model. However, this relatively small contribution of BMI and weight-related behaviors to the relationship between acculturation and diabetes prompts consideration of other bio-behavioral mechanisms underlying this association. The stressful impact of acculturation on immigrants, or “acculturative stress” (26), may cause adverse metabolic consequences unrelated to BMI that contribute to the positive association found here between acculturation and diabetes. Experimental evidence from both animal and human studies has linked the perception of stress with physiologic responses that are harmful to health (27). This physiologic stress response, which involves activation of the hypothalamic-pituitary axis and chronic inflammation, has been implicated in the development of type 2 diabetes (28). A recent NHANES study reported unhealthier inflammatory and metabolic profiles, including HbA_1c_, among US-born versus foreign-born Mexican Americans (29).

A recent study revealed significant heterogeneity in diabetes risk among Latino subgroups (20), which may stem in part from their different acculturation experiences in the United States. Future research should explore the relationship between acculturation and diabetes among all major Latino subgroups separately. Longitudinal studies of Latinos should explore biologic correlates of acculturation — including physiologic markers of psychological stress — and their associated diabetes risk, which may imply a causative role for acculturation in diabetes incidence. Large survey studies should include standardized, validated instruments to measure acculturation, which would facilitate interpretation of study findings and comparisons across studies. Qualitative research in this area may serve as an important complement to epidemiologic studies by exploring the process of acculturation in more depth than is possible using standard survey questions. Using such knowledge about Latino culture in health care interactions may improve providers’ efforts to address diabetes in the clinical setting (30). Therefore, future studies that examine the social context surrounding acculturation and diabetes in US Latinos may provide unique insights into how to prevent and treat diabetes in this high-risk population.
